# Supplementary material for: Transcriptomic Insights into Mechanisms of Early Seed Maturation in the Garden Pea (Pisum sativum L.)
Source: Cells. 2020 Mar 23;9(3):779. doi: 10.3390/cells9030779 (PMC7140803; doi:10.3390/cells9030779)

Correlation Network on module data (dmap)

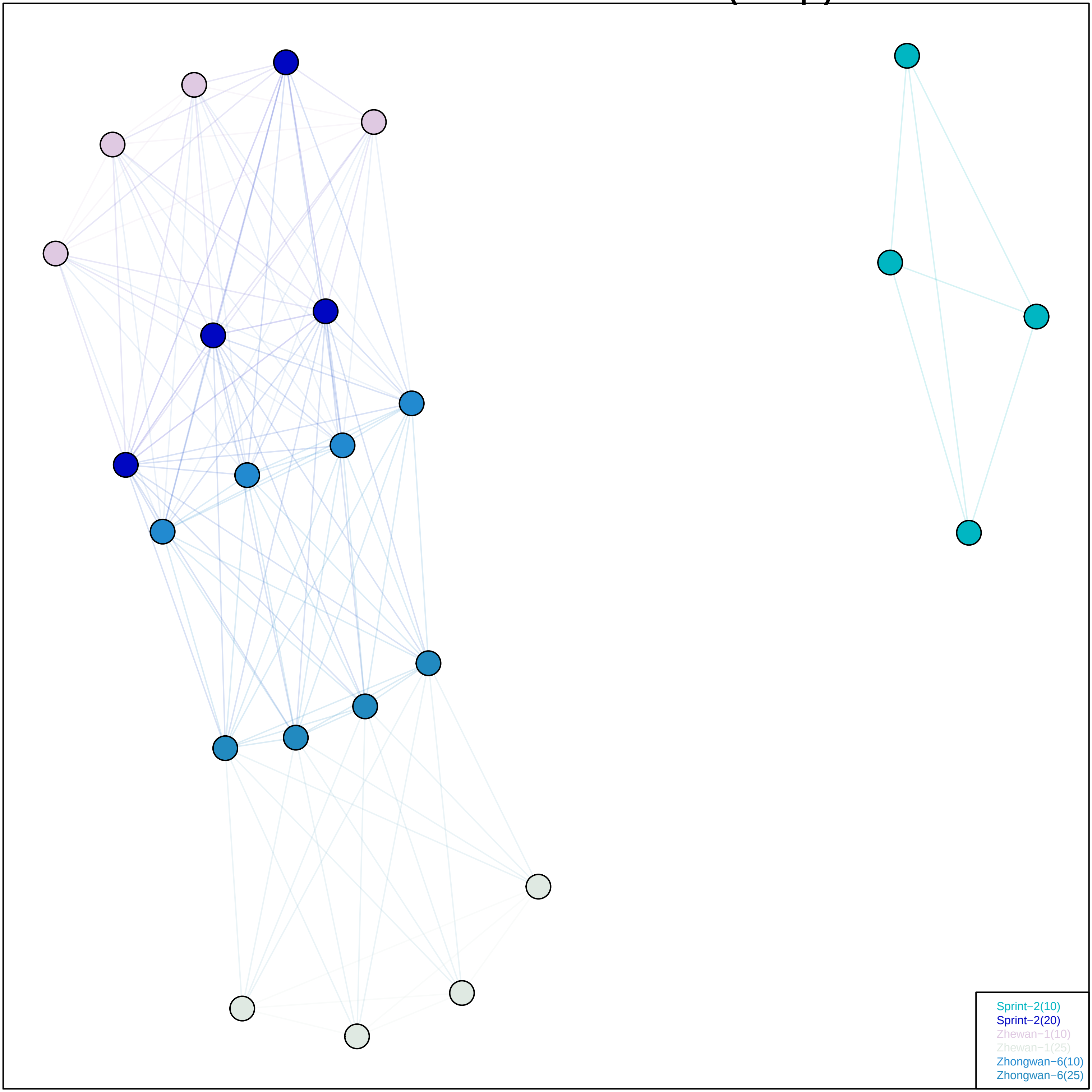

Correlation Network & silhouette scores on module data (dmap)

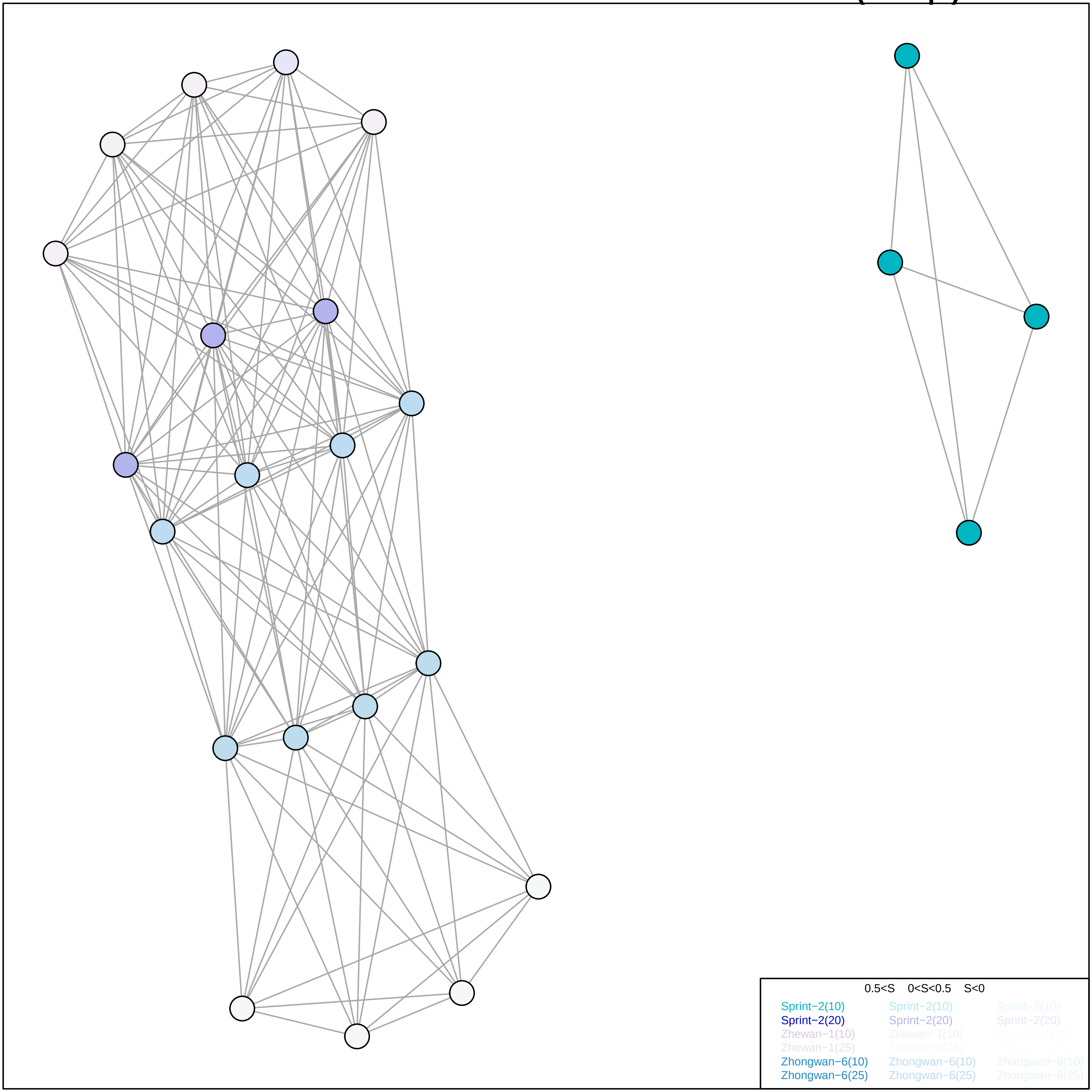

Correlation Network on module data (dmap)

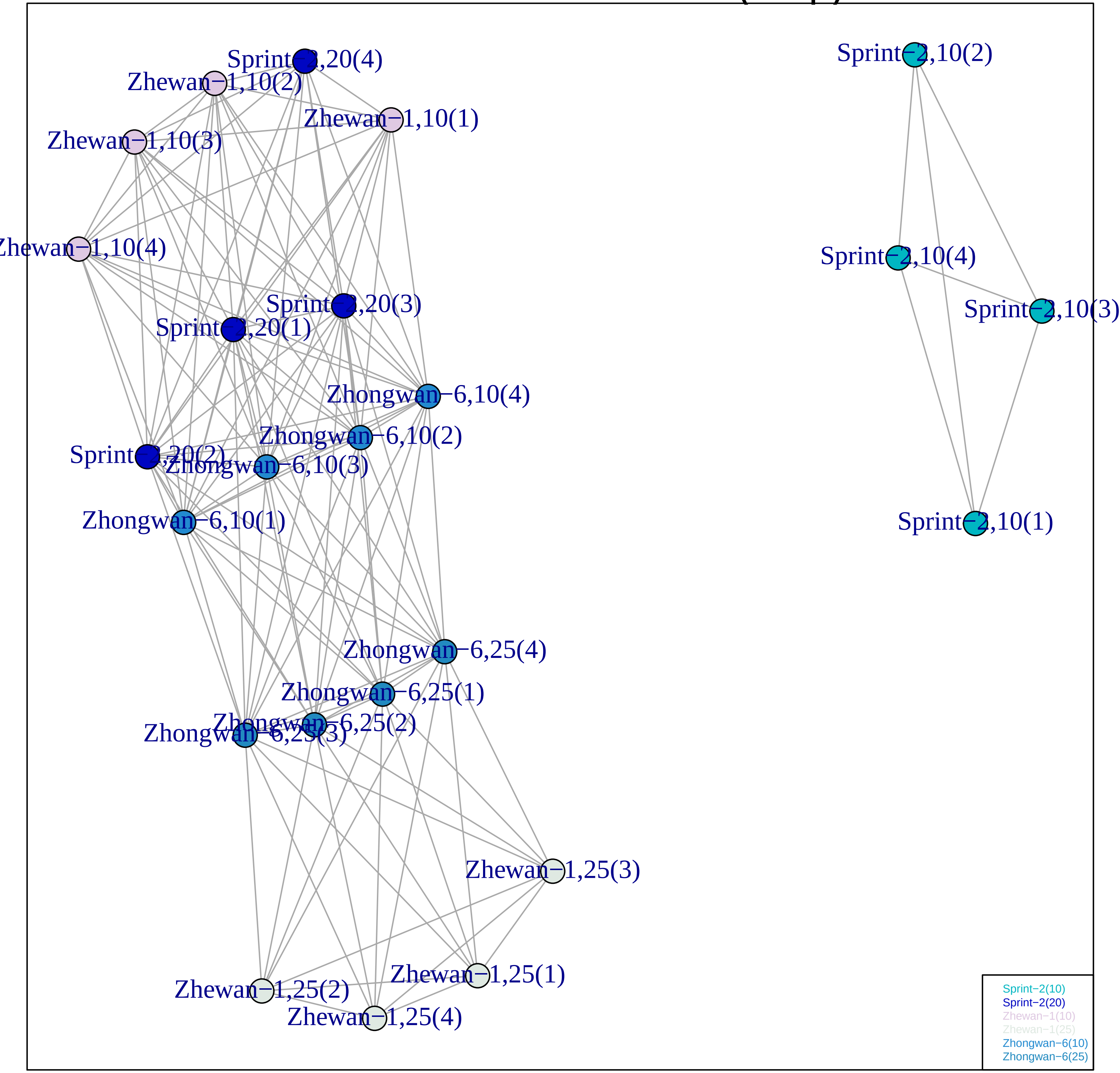

Correlation Network on metagene data

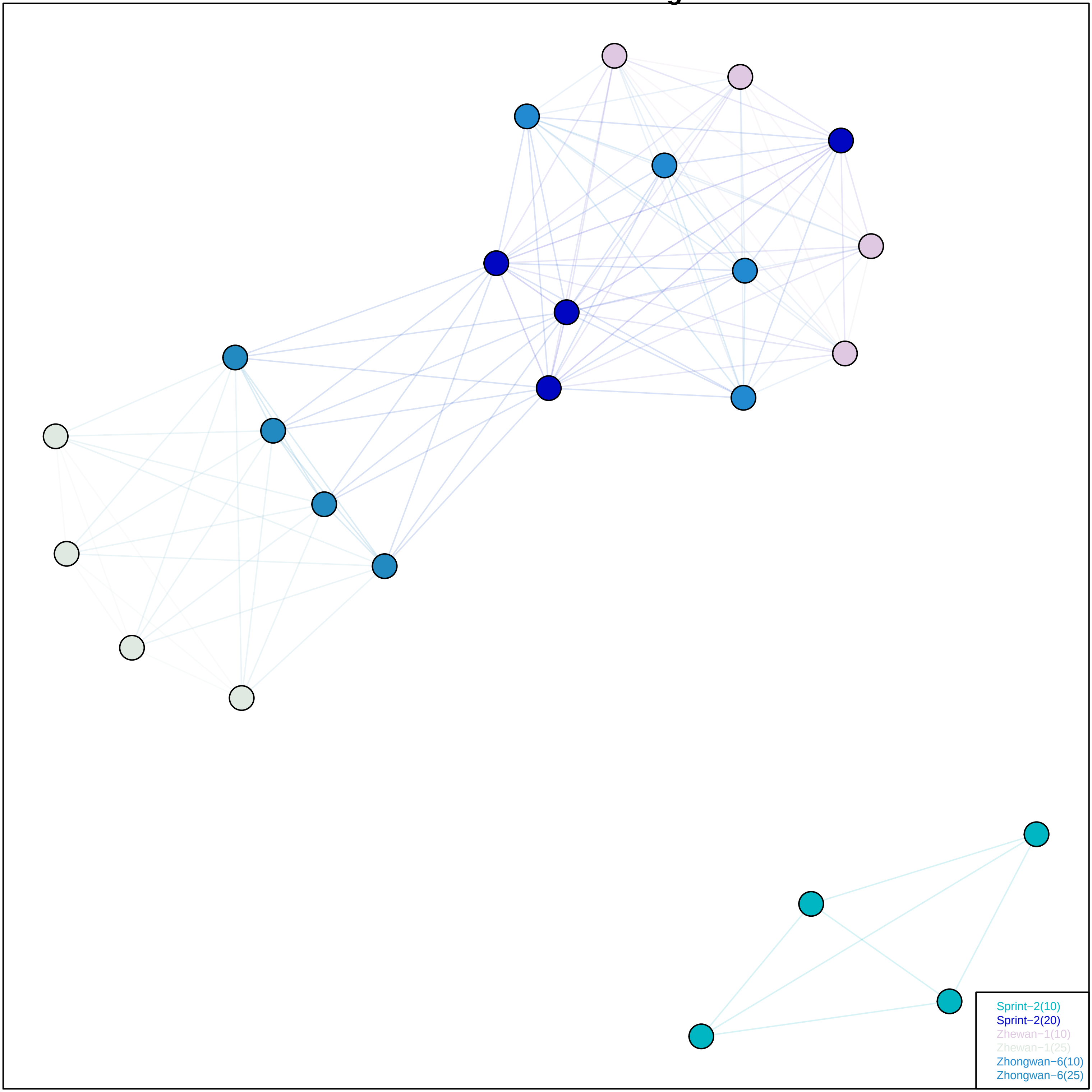

Correlation Network & silhouette scores on metagene data

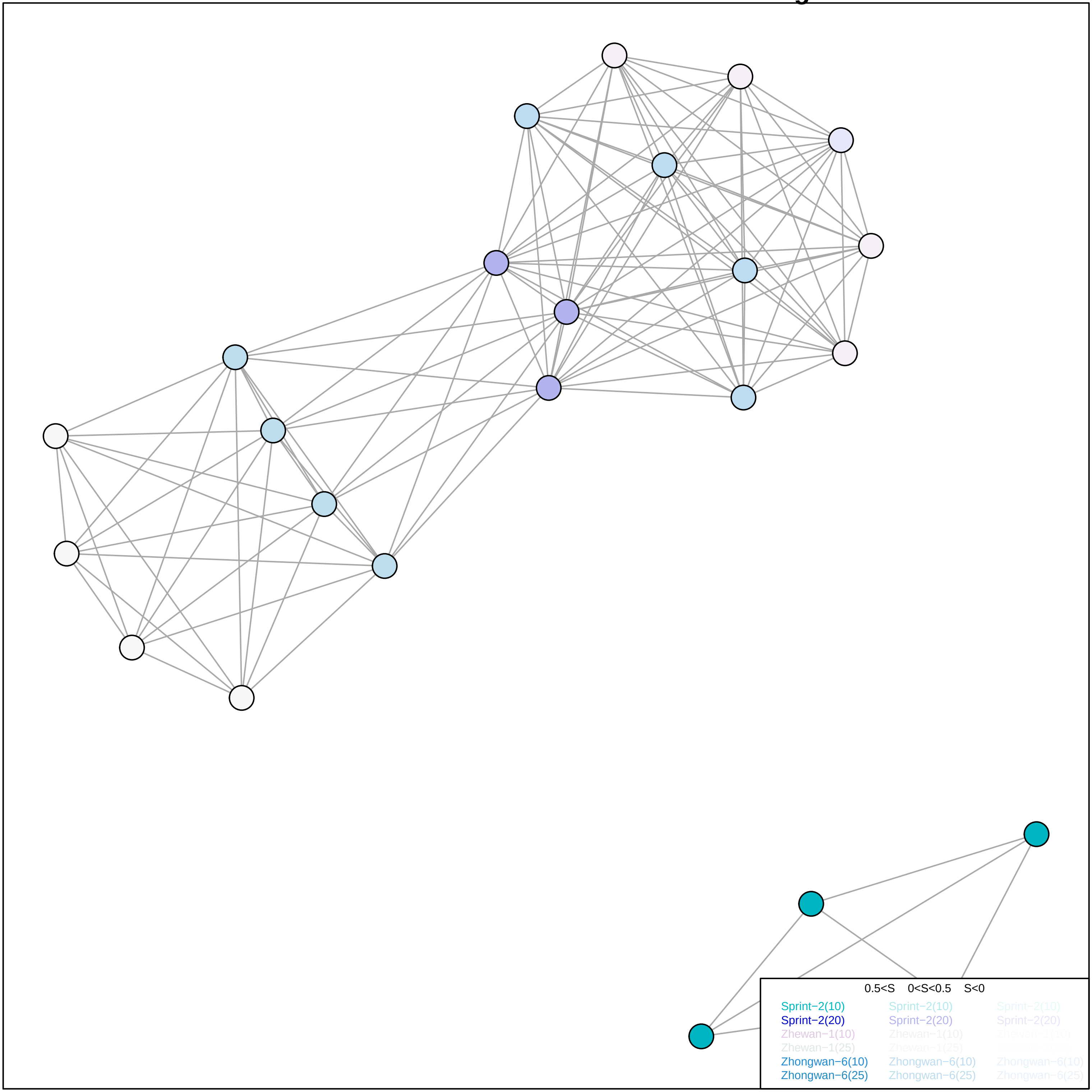

Correlation Network on metagene data

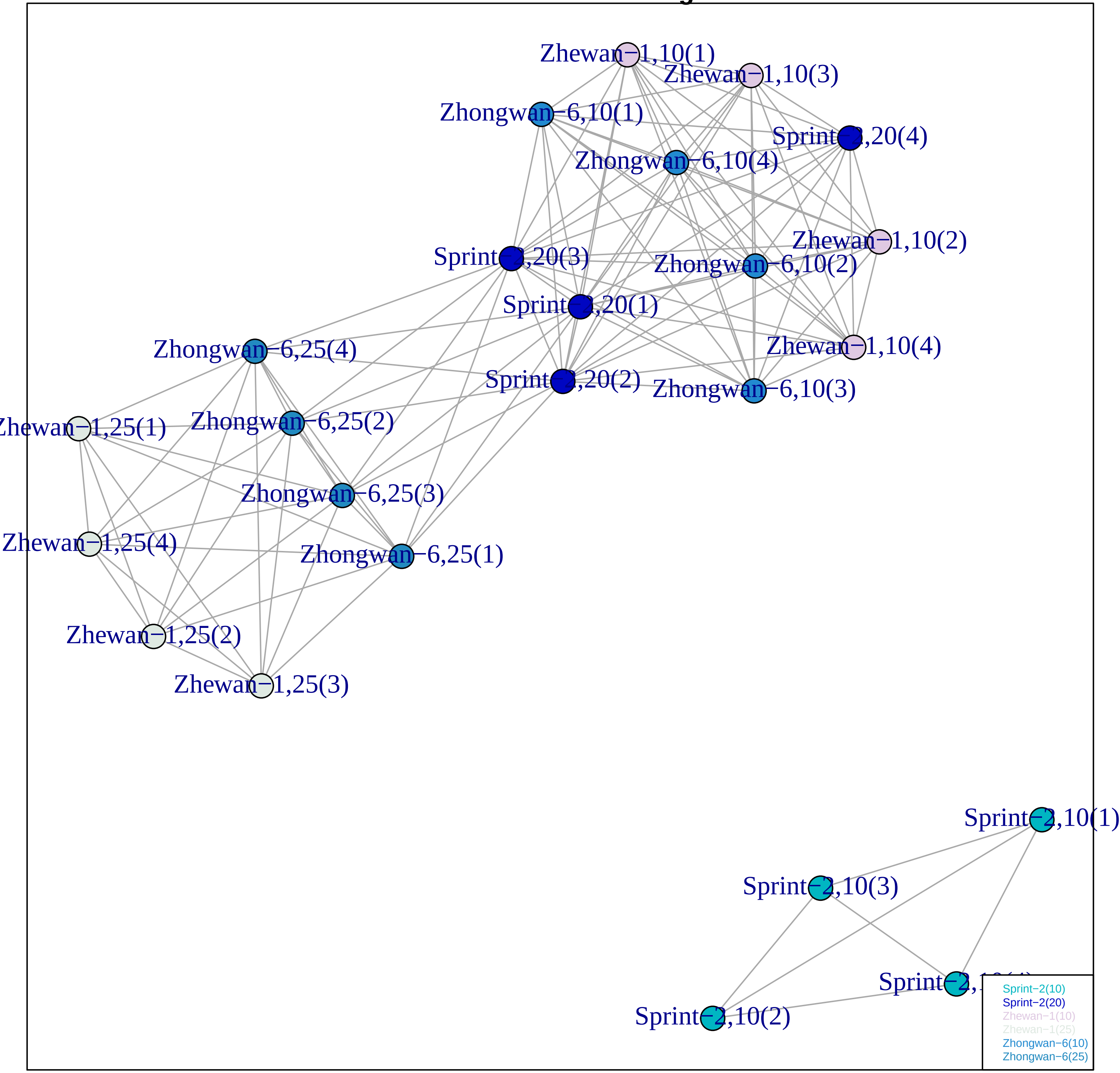

Supplement: Supplementary file 1 [file cells-09-00779-s001.zip › Supplementary materials/FileS2.zip!/FileS1/Sample Similarity Analysis/Correlation Network.pdf]
